# Impact of the Host Microbiome on Vaccine Responsiveness: Lessons Learned and Future Perspective

**DOI:** 10.1021/acs.biochem.2c00309

**Published:** 2022-08-22

**Authors:** Giuseppe Stefanetti, Dennis L. Kasper

**Affiliations:** † Department of Biomolecular Sciences, University of Urbino Carlo Bo, 61029 Urbino, Italy; ‡ Department of Immunology, Blavatnik Institute, Harvard Medical School, Boston, Massachusetts 02115, United States

## Abstract

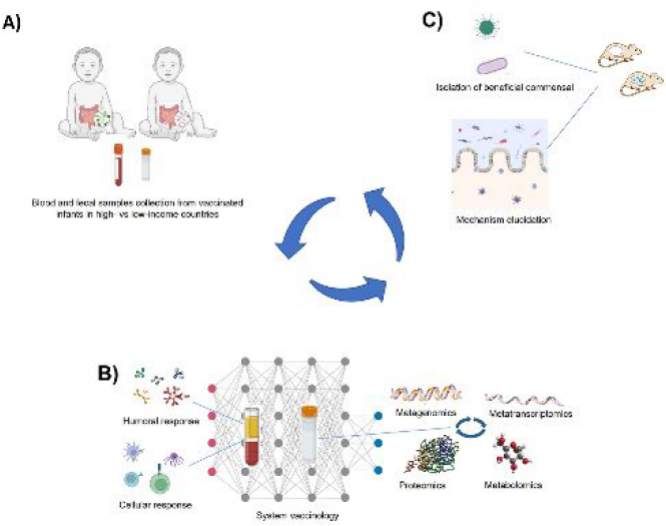

Vaccination shows high variability in the elicited immune responses among individuals and populations for reasons still poorly understood. An increasing number of studies is supporting the evidence that gut microbiota, along with other interplaying variables, is able to modulate both humoral and cellular responses to infection and vaccination. Importantly, vaccine immunogenicity is often suboptimal at the extremes of age and also in low- and middle-income countries (LMICs), where the microbiota is believed to have an important role on immune responses. Still, contrasting findings and lack of causal evidence are calling for sophisticated methodologies to be able to overcome scientific and technical challenges to better decipher the immunomodulatory role of microbiota. In this perspective, we briefly review the status of the vaccine field in relation to the microbiome and offer possible scientific approaches to better understand the impact of the host microbiome on vaccine responsiveness.

## Introduction

### Need for Highly Effective Vaccines

Vaccinations have had an unparalleled impact on global health.^1^ Vaccines have great potential to further improve health in the poorest countries of the world where infectious diseases account for roughly half of the deaths.^2^ Interestingly, vaccine induced immune responses are highly variable between individuals and populations in different regions of the world with longstanding concerns related to nonresponder cohorts. Vaccine-induced antibody levels have significant variability between individuals (e.g., ∼100-fold for the inactivated seasonal influenza vaccines, ∼40-fold for pneumococcal and *Haemophilus influenzae* type b (Hib) conjugate vaccines). Cellular immune responses are also affected, as demonstrated by the ∼100-fold variability in cytokine response elicited by the Bacille Calmette-Guerin (BCG) vaccination for tuberculosis.^3,4^ Importantly, vaccine immunogenicity is mainly impaired in populations at the highest risk for disease, including vaccine recipients in low- and middle-income countries (LMICs),^5^ infants,^6^ and elderly.^7^ Many factors influence the immune response to a specific vaccine,^3^ including schedule, intrinsic host factors (e.g., age, sex, genetics, and comorbidities), perinatal factors (e.g., gestational age, birth weight, breastfeeding, maternal infections, and antibodies), and extrinsic factors (e.g., trained immunity, preexisting immunity, microbiota, infections, antibiotics use). In addition, environmental factors (e.g., geographic location, season, family size, and toxins), behavioral factors (e.g., smoking, alcohol consumption, exercise, stress and sleep), and nutritional factors (e.g., body mass index, nutritional status, micronutrients, and enteropathy) also influence how individuals respond to vaccines. Understanding the influence of these variables on vaccine responses and designing new interventions to strengthen the immune system’s response to vaccines is of the utmost importance.

### Impact of Microbiota on Immunity to Vaccination

Evidence indicates that the gut microbiota is variable between individuals^3^ and over the course of life.^8^ The microbiota also varies between different populations at different geographic locals and on different diets.^9^ These are important factors modulating the immune responses to vaccination.^4,10^ The makeup of the microbiota has been correlated with the vaccination outcome and with other factors such as age, diet, metabolism, and chronic infection.^11^ The microbiota of humans contains many times more genes than host-encoded genes,^12^ and the gastrointestinal tract is the largest reservoir for microbes, the so-called “second genome”. The microbial community of the host has been shown to be critical in shaping physiology and immune responses,^13−15^ regulating autoimmunity and allergy,^16−19^ preventing HIV infection,^20^ and modulating anti-PD1 cancer immunotherapy.^21^ While most of the evidence in support of the microbiome’s impact on response to vaccination comes from work in mouse models, several observational clinical cohort and interventional studies have investigated, with conflicting findings, the possibility that gut microbiota can modulate immune responses to vaccination.^4,8^ The possible role of microbiota in modulating immune responses to vaccinations is of particular concern in LMICs, where extensive use of antibiotics in neonates and infants can cause long lasting microbiota changes.^22^

## Lessons Learned

### Correlative Evidence from Clinical Studies

Associations between infant microbiota and vaccine responsiveness have been reported in several observational clinical studies.^23−26^ Immune responses to oral vaccines (such as oral rotavirus vaccines (ORVs) and oral polio vaccines (OPVs)) are lower in LMICs when compared with high-income countries, and therefore these interventions have been the focus of many studies investigating the role of microbiota in modulating immune responses. Of note, infant studies in Ghana^23^ and Pakistan^24^ reported a significant association between the fecal microbiota and the response to ORVs. Harris et al. reported a nested case-control study showing that microbiome composition of Ghanian infants was different between ORV responders and nonresponders.^23^ The microbiota of Ghanaian responders was more like that of Dutch infants who were assumed to respond well to vaccinations. In this study, an increased relative abundance of *Streptococcus bovis* was significantly correlated with an enhanced response to vaccination, whereas the relative abundance of *Bacteroides* and *Prevotella* species were negatively correlated.^23^ In a similar study conducted in Pakistan, the ORV response was correlated with a higher relative abundance of bacteria belonging to Clostridium cluster XI and Proteobacteria.^24^ Both studies reported an increased ratio of *Enterobacteriaceae* to *Bacteroides* species in vaccine responders. In contrast, other studies in infants in both India and Nicaragua did not find any significant associations between the fecal microbiota and responses to ORVs.^27,28^ However, in these latter two studies, the authors speculated that infants might have harbored a microbiota that was inhibitory to rotavirus vaccine replication. As for OPV, a study in China found that the relative abundance of *Bifidobacterium* in the infant fecal microbiota was correlated with increased poliovirus-specific IgA responses.^25^ By contrast, another study conducted in infants in India^29^ did not find any significant differences in the relative abundances of specific taxa between responders and nonresponders to OPV. Enteric viruses were shown to have a greater impact on OPV response than the bacterial microbiota, with recent enterovirus infections having a greater inhibitory effect than persistent infections, a finding that suggested a possible role also for the host virome. Interestingly, in both studies,^25,29^ greater microbiota diversity was associated with poor vaccination responses, but it is also possible that this is only a marker of exposure to enteric infections. In a prospective observational study, the relative abundance of *Bifidobacterium* in early infancy has also been found to be significantly associated with CD4^+^ T cell and antibody responses to several parenteral vaccines assessed at 2 years of age,^26^ indicating that an increase in the abundance of *Bifidobacterium* may enhance the protective efficacy of vaccines and suggesting that microbiota might also modulate responses to nonorally administered vaccines. Interestingly, a unique immunomodulatory role for *Bifidobacteria* has been evidenced in regulating the response to checkpoint inhibitor immunotherapy in mice, which could suggest a potential role for this bacterium also in regulating vaccine responses.^50,51^

### Antibiotic Interventional Studies

An increasing number of conflicting observational and interventional studies have investigated the role of antibiotic-driven perturbations of the gut microbiota in vaccine responses. In Dutch adults, narrow-spectrum but not broad-spectrum antibiotics, administered to reduce bacterial-derived enteropathy associated with impaired oral vaccine immunogenicity, resulted in more effective day-7 antirotavirus IgA boosting. An increased proportion of volunteers with more than a 2-fold increase in anti-rotavirus IgA titer and enhanced ORV antigen shedding was interpreted as an indication of better replication and response to vaccination.^30^ Interestingly, as in the Ghanian infant study on response to ORVs conducted by the same group,^23^ an increase in the ratio of Enterobacteriaceae to Bacteroides in vaccinated adults was associated with enhanced IgA boosting.^30^ However, the same study also reported that antibiotics did not affect either the pneumococcal polysaccharide (no adjuvant) or the adjuvanted tetanus toxoid responses.^30^ An investigation targeting Indian infants showed that antibiotics did not improve the immunogenicity of OPV, despite reducing biomarkers of enteropathy and pathogenic intestinal bacteria.^31^

Remarkably, a systems vaccinology approach has been recently used to comprehensively assess the impact of broad-spectrum antibiotics on the innate and adaptive immune response to tetravalent inactivated influenza vaccination.^13^ Broad spectrum antibiotics were administered to groups of healthy young adults before and after vaccination. The antibiotics resulted in a significant reduction in gut bacterial numbers and diversity but had no significant impact on antibody responses. Importantly, these subjects had pre-existing humoral immunity with high influenza-specific microneutralizing antibody titers before vaccination. Remarkably, analysis of the vaccine responses in a second trial of subjects with low pre-existing antibody titers revealed a significant impairment in both neutralizing and binding antibodies. Antibiotic treatment led to a significant reduction in the IgG1 and IgA antibody response toward the H1N1 strain as well as to a decrease in the antibody neutralization to the same strain. This finding suggests that microbiota immunomodulation of adaptive response to vaccination is playing a minor role in the presence of pre-existing humoral immunity. Interestingly, the impairment in IgG1 and IgA responses was only observed against one of the three influenza strains contained in the vaccine, the H1N1 strain, and not against the H3N2 or B strains. This outcome was hypothesized to be related to different prior exposure to the different influenza strains, which could lead to a different threshold of memory responses.^22^

In addition to influencing the adaptive response, the analysis of transcriptional signatures revealed that treatment with antibiotics led to altered innate immune responses. Several gene expression programs associated with transcription factors playing key roles in inflammatory responses were up-regulated under antibiotic treatment. Interestingly, the same transcriptional modules were increased in healthy elderly subjects immunized with the seasonal influenza vaccine,^32^ a result indicating the possibility that long-term usage of antibiotics may lead to a chronic stage of low-grade inflammation and contribute to the pathogenesis of age-associated diseases. Antibiotic administration also led to divergent metabolic trajectories, highly correlated with immune signaling. Among other changes, a reduction in bile acids, such as lithocholic acid (LCA), was observed.^13^ Given the role of secondary bile acids in suppressing inflammation, a potential mechanism by which the microbiota can regulate secondary bile acid production, and consequently, inflammatory responses in humans affecting therefore vaccine response could be envisioned.^22^

### Microbiota-Targeted Interventional Studies

Intervention studies targeting possible alterations of the microbiota, such as diet, prebiotics, probiotics, symbiotics, fecal microbiota transplant (FMT), and small-molecule drugs, are increasingly being conducted. Research suggests that modulating the microbiota may not only improve symptoms but also can have a significant effect on reducing life-threating disease, such as for the prevention of sepsis and necrotizing enterocolitis in infants.^33,34^ Probiotics, microorganisms introduced into the body for their beneficial qualities, have been effectively used to prevent important diseases such as necrotizing enterocolitis^35^ and acute diarrhea.^36^ Synbiotics, mixtures of probiotics and prebiotics (i.e., nondigestible food ingredient that promotes the growth of beneficial microorganisms in the intestines), have shown to be able to prevent sepsis among infants in rural India^37^ and to improve efficacy of oral cholera vaccination in a mouse model of childhood undernutrition.^38^ However, studies of probiotic impact on boosting vaccine responses in infants and adults have reported variable results (estimated rate of observed beneficial effect is around 50%). This result was shown to be dependent on multiple variables including the immunizing antigen, the strain of probiotic and the geographical region of the study. Unfortunately, most of the studies reported so far have important limitations that do not allow for direct comparison and there are no real generalizations of results that can be made. These limits include sample size, differences in the probiotic strains investigated, and the administration schedule. Finally, the lack of studies directly focusing on participants with already disrupted microbiota who are those most likely to receive benefit is another complicating variable.^4,39^

### Evidence from Preclinical Studies and Proposed Mechanisms of Immunomodulatory Action

Numerous investigations have demonstrated a role for microbiota in modulating immune responses to both infection^40−44^ and vaccination.^14,15,40,45^ Germ-free (GF) and/or antibiotic-treated animals are often used to study the effect of the microbiota on the development and homeostasis of the host immune system and on the immune response to the vaccination.^46^ In one study, both antibiotic-treated and GF mice showed enhanced IgG and IgA responses to an orally administered mouse rotavirus strain.^40^ In contrast, following immunization with ovalbumin, germ-free pups as well as pups born to antibiotic-treated dams showed reduced IgG responses when compared to immunized microbiota-competent controls. However, these differences were modest and depended upon the immunization schedule.^45^ In another study, the response to nonadjuvanted influenza vaccine was found to be impaired in GF, antibiotic-treated, and Toll-like receptor 5 (Tlr5)-deficient mice,^15^ suggesting that TLR5-mediated sensing of flagellin produced by the microbiota could act as a natural adjuvant for nonadjuvanted vaccines.

More recently, age has been shown to be particularly relevant to vaccine responses, reinforcing the concept that microbiota-dependent immunomodulatory effects may be more important in the early stages of life.^14^ Dams were exposed to antibiotics prenatally. As a result of maternal treatment, antigen-specific IgG responses to live attenuated BCG and four adjuvanted vaccines in young mice were found to be significantly impaired.^14^

Overall, the mechanisms by which the microbiota modulate immune responses to vaccination are not well understood, but it is safe to assume that different pathways are acting contemporaneously. Several immunoregulatory mechanisms have been proposed,^4^ including (1) the natural adjuvant hypothesis, i.e., the ability of microbiota-associated immunomodulatory molecules, such as flagellin^15^ and peptidoglycan,^47^ to modulate vaccine responses by stimulating pattern recognition receptors (PRRs) on antigen-presenting cells (APCs), such as toll-like receptors (TLRs)^15^ and NOD2;^47^ (2) microbiota-induced antigen presentation by DCs,^48,49^ such as the regulation of type I INF expression by plasmacytoid DCs (pDCs), which can instruct a specific metabolic and epigenomic state in conventional DCs (cDCs) enhancing T cell priming;^49^ (3) immune activation by microbiota-derived metabolites, such as short-chain fatty acids (SCFAs), which have been shown to increase B cell metabolism to support antibody production and to increase expression of genes involved in class switching and plasma cell differentiation;^43^ and (4) microbiota-derived B cell and T cell epitopes, which could potentially cross-react with pathogen-encoded epitopes and alter the responses to vaccination.^4^ Potential redundancies between these and other commensal-dependent pathways and the context-dependent role of specific microbiota composition complicate the study of microbiota-dependent immunomodulatory mechanisms of action.

## Challenges Ahead and Open Questions

**Figure 1:**
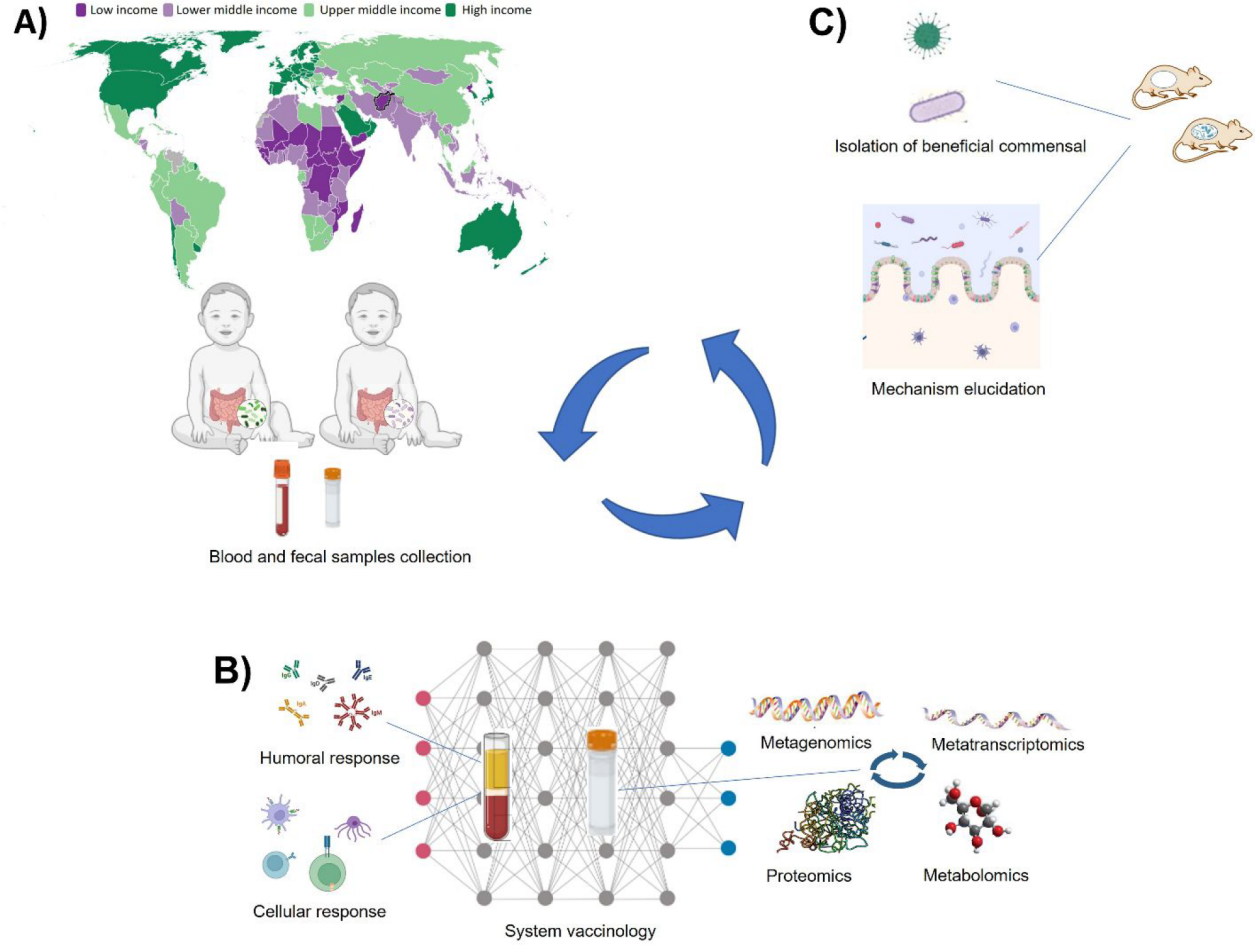
Deciphering microbiota-dependent immunomodulation of vaccine responses. Powered-designed interventional studies focusing on the early stages of life in HICs vs LMICs (A) could allow the identification of groups of individuals of interest for further analysis by systems vaccinology approaches (B) to define the microbiota-dependent cellular and molecular changes that occur in response to vaccination. Selected human microbiota could then be transferred to gnotobiotic animals (C) for mechanistic evaluation and for screening of immunomodulatory taxa. Classifications of income status are based on World Bank data. https://datatopics.worldbank.org/world-development-indicators/the-world-by-income-and-region.html.

Several complicating factors may explain the current lack of critical knowledge regarding the microbiota-dependent immunomodulation of vaccine responses. While sophisticated mechanistic studies in mice have demonstrated the impact of microbiota on both physiology and pathology, their relevance in humans is unclear with most evidence coming from correlative studies. Causal evidence for the role of the microbiota in modulating human physiology and susceptibility to disease is still scarce and hampered by scientific and technical challenges. We report below some of the current challenges in this field and comment on possible actions to enable a better understanding of the role of microbiota in the response to vaccination (Figure 1).

### Powered-Designed Interventional Studies Focusing on the Early Stages of Life

Vaccine immunogenicity is often suboptimal in populations at high risk for acquiring infectious diseases, including neonates, infants, elderly, and LMICs regions. The evolution of the microbiome in newborns coincides with a crucial maturation period for the immune system. Since the earliest stages of life coincide with the time the first vaccinations are given, it is highly probable that during this period the imprinting of the microbiota can have significant long-term and permanent effects on the development of immune responses.^52^ A critical “window of opportunity” exists in early childhood where the microbiota could have a major effect on the modulation of vaccine responses. Impaired responses to five licensed infant vaccines was reported in infant, but not adult, mice when exposed to antibiotics.^14^

Previous findings have also shown the importance of a “weaning reaction” to microbiota for immune ontogeny and to reduced susceptibility to colitis, allergic inflammation, and cancer later in life.^53^ Furthermore, a greater impact of microbiota on antibody responses in humans with low levels of pre-existing immunity has been observed,^13^ suggesting a more pronounced immunomodulatory role with the priming doses of vaccines administered at less than 6 months of age, rather than on booster responses.

Associations between the infant microbiota and responses to vaccination have been reported in several observational clinical studies.^23,24^ Nevertheless, most of the antibiotic-based interventional investigations performed so far have assessed the impact of antibiotics on vaccine responses in adults and were limited by a small sample size and a short time window between antibiotic-treatment and vaccine administration.^4^ LMICs are also a target of great interest, as widespread use of antibiotics in these populations, mainly neonates and infants, is associated with long lasting microbiota changes which could affect the immune development and the response to vaccination.^22^ The composition of the gut microbiota is highly variable between individuals, particularly between Westernized and non-Westernized populations and high-income countries (HICs) vs LMICs. Given the differences in the microbiota, it is foreseeable to conceive that a commensal/probiotic beneficial in infants in HICs will not have the same effect in LMICs. Therefore, well-powered randomized controlled trials are needed to evaluate the beneficial effects of microbiota in modulating the response to vaccination in these target populations.

### Interdependency of Microbiota-Vaccine Response Association

The microbiota is only one of the interdependent determinants associated with the magnitude of vaccine responses. Other factors, including genetic and environmental variables (e.g., diet, stress, presence of infections, age,...), shape the physiological state of individuals and their response to antigen stimulation.^11^ Several studies that aimed to assess the impact of the microbiome on vaccine response have been conducted in geographically different populations, as in LMICs and HICs, with different microbiome compositions.^23,30^ Furthermore, the administration of antibiotics before vaccination can generate potential off-target effects, which could impact the immune responses and influence the interpretation of the study.^30^

This complexity calls for a sophisticated systems vaccinology approach to define the microbiota-dependent cellular and molecular changes that occur in response to vaccination. Systems vaccinology, i.e., the application of systems biology methods to analyze vaccine responses, has emerged with the need to integrate large sets of data coming from new high-throughput technologies using mathematical and computational modeling. Systems vaccinology approaches have delivered useful information about adjuvants and innate immunity, and they are undoubtedly necessary to help disentangle the complexity of microbiota-immune response interactions.^54^ Next generation sequencing (NGS) approaches (e.g., metataxonomics, metagenomics, metatranscriptomics, and metabolomics), used to characterize the microbiota composition, can be integrated with advanced immunological profiling (e.g., multiparametric flow cytometry, transcriptomic analysis, system serology) by systems biology approaches to better correlate the influence of the intestinal microbiome on vaccine responses. Given the complexity of this interaction, systems-level integrated studies can help identify microbiota-associated molecular and cellular signatures associated with protective immunity.

Some current limitations need to be overcome to fully exploit the potential of system vaccinology. Several areas require attention including (1) the identification of predictors of immune responses in tissues, (2) challenges that face current proteomic technologies such as complex sample preparation, reproducibility, limited dynamic range, and detection of post-translational modifications;^55,100^ (3) the development of robust signatures of protective immune responses capable of predicting vaccine efficacy in clinical settings; and (4) the need to translate the data generated into meaningful understanding about the mechanisms of microbiota-induced immune regulation to vaccine responses. Achieving these capabilities require fruitful collaborations between scientists with different expertise (including systems biologists, microbiologist, bioinformaticians and immunologists).^55^

### Identification of Microbiome Targets Correlating with Vaccine Immunogenicity

An increasing number of observational studies in infants have acknowledged associations between specific commensal phyla and families with immune responses to vaccination.^26^ Identifying clinically relevant microbial taxa with immunomodulatory potential will be essential to prove the causal relationship and to elucidate mechanisms of action. In this regard, the application of newer sequencing technologies, such as shotgun metagenomics, which comprehensively sample all genes in all organisms present in a given complex sample, could allow for higher resolution up to species- and strain-specific levels.^4^ This is quite relevant as the ability of microbes to induce similar immunophenotypes is unrelated to their phylogeny, with distant phyla capable of inducing similar immunomodulatory effects while different species from the same genus can induce opposing ones.^56^

Gnotobiotic models, including GF and antibiotic-treated mice, have provided key insights on the microbiota-immune system interplay,^57^ including the identification of commensals responsible for the intestinal immune system development,^58,59^ and are essential to explore how host–microbe interactions modulate vaccine responses.^60^ Our lab has previously characterized the immunomodulatory effects of over 60 different human gut-derived bacteria.^56^ Germ-free (GF) mice were monocolonized with a commensal microbe followed by immunoprofiling and microarray analysis of the immune system. Most microbes exerted several specialized, complementary, and redundant transcriptional and immunomodulatory effects which were, remarkably, independent of microbial phylogeny. Similar studies could pave the way for the analysis of microbiota-vaccine response interplay using microbiota samples of clinical relevance. Causal relationships between microbiota and vaccine responses could be established by transferring selected human microbiota (HMB) to GF mice for mechanistic evaluation and screening of immunomodulatory taxa. After mono- or selected-colonization of GF mice with HMB, extensive and unrestricted immunophenotyping and transcriptomics, as were previously performed^56^ before and after vaccination, could lead to key information regarding the immunomodulatory roles of key identified taxa. Ultimately, one could evaluate if the selected immunomodulatory taxa are able to also elicit an immunomodulatory role in the specific-pathogen free (SPF) setting. Although the use of GF or selected-microbiota models does not fully represent the complex interactions that occur within the microbiota-competent environment of conventional mice, this deconvolution is necessary to control complexity and interdependency of additional variables (e.g., genetics/use of littermates, age, diet, metabolic status, external environment) associated with the immune response.

## Conclusion

Further efforts are needed to understand the microbiome-immune interaction and how this influences vaccine response. Although the gut microbiota is known to modulate both B cell and T cell responses to vaccination, additional work is necessary to move from correlation to causation by defining clinically relevant microbiota-dependent immunomodulatory mechanisms. Areas for improvement include (Figure 1): (1) designing appropriate interventional studies focused on early life across a broad geographical and socioeconomic spectrum; (2) using systems vaccinology approaches to help navigating through the complexity of microbiota-immune response interactions; and (3) identifying immunomodulatory taxa of clinical relevance to prove a causal relationship and to elucidate the mechanisms of action on the immune response by using gnotobiotic models. Potentially, these advances could pave the way for the discovery of important signatures and pathways leading to microbial immune-enhancing interventions of general and vaccine-related importance.
